# Retraction: *Brassica juncea* Lines with Substituted Chimeric GFP-CENH3 Give Haploid and Aneuploid Progenies on Crossing with Other Lines

**DOI:** 10.3389/fpls.2018.00043

**Published:** 2018-01-16

**Authors:** Frontiers Editorial Office

The journal retracts the 6 January 2017 article cited above. Following concerns identified post-publication, the article was examined by the Chief Editors, confirming image duplication in Figure 5 and discrepancies in the data sets presented which precludes a Correction. The Field Chief Editor therefore concluded that the article warranted retraction. The authors agree to the retraction and the notice.

